# Tetrahedral Gray Code for Visualization of Genome Information

**DOI:** 10.1371/journal.pone.0086133

**Published:** 2014-01-27

**Authors:** Natsuhiro Ichinose, Tetsushi Yada, Osamu Gotoh

**Affiliations:** 1 Department of Intelligence Science and Technology, Graduate School of Informatics, Kyoto University, Yoshida-Honmachi, Sakyo-ku, Kyoto, Japan; 2 Department of Bioscience and Bioinformatics, Kyushu Institute of Technology (KIT), Kawazu, Iizuka, Fukuoka, Japan; 3 Computational Biology Research Center (CBRC), National Institute of Advanced Industrial Science and Technology (AIST), Aomi, Koto-ku, Tokyo, Japan; Swiss Institute of Bioinformatics, Switzerland

## Abstract

We propose a tetrahedral Gray code that facilitates visualization of genome information on the surfaces of a tetrahedron, where the relative abundance of each 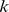-mer in the genomic sequence is represented by a color of the corresponding cell of a triangular lattice. For biological significance, the code is designed such that the 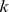-mers corresponding to any adjacent pair of cells differ from each other by only one nucleotide. We present a simple procedure to draw such a pattern on the development surfaces of a tetrahedron. The thus constructed tetrahedral Gray code can demonstrate evolutionary conservation and variation of the genome information of many organisms at a glance. We also apply the tetrahedral Gray code to the honey bee (*Apis mellifera*) genome to analyze its methylation structure. The results indicate that the honey bee genome exhibits CpG overrepresentation in spite of its methylation ability and that two conserved motifs, CTCGAG and CGCGCG, in the unmethylated regions are responsible for the overrepresentation of CpG.

## Introduction

**Figure 1:**
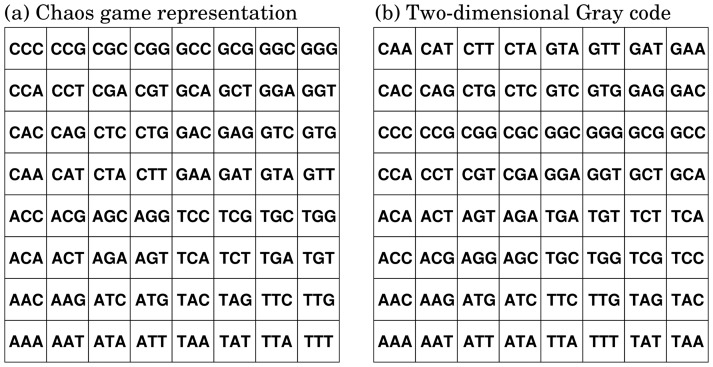
Transformations of CGR (a) and 2D Gray code (b) of trimers.

One of the first steps in exploring the huge amount of information contained in genomes is content visualization of short nucleotide sequences of a fixed length of 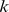 (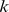-mers). The landmark study of such visualization is Jeffrey's chaos game representation (CGR) [1]. CGR is a transformation between a DNA sequence and a position in a unit square. All sequences having the same prefix are transformed into the corresponding box as shown in Fig. 1(**a**). When all suffixes in a genome are transformed, its frequency distribution in the unit square represents the content information. Because of the simplicity and visibility of CGR, several applications and extensions have been proposed, such as extensions to protein or arbitrary sequences [2], [3], alignment-free comparisons of genomes [4], fractal analysis [5], [6], and analysis of Markov properties [7]. For the purpose of information visualization, however, CGR has a serious drawback in that adjacent 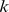-mers can be completely different from each other, *e.g.*, CTT and GAA in Fig. 1(**a**). Thus, physical proximity in CGR does not necessarily indicate similarity of the corresponding 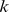-mers.

Besides CGR, we can consider various transformations of DNA sequences. What transformation is most informative for DNA sequence analysis? One of the best candidates is the Gray code [8]. The Gray code is originally defined as an ordering of binary numbers in which adjacent numbers differ from each other by only one bit. We can easily extend it to quaternary numbers corresponding to DNA sequences [9], *i.e.*, the Hamming distance between the adjacent 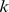-mers is always one in the code. Such DNA Gray code has been applied to motif discovery [10].

Another candidate for transformation is the de Bruijn code (or cycle) [11]. In the de Bruijn code, adjacent 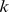-mers have the shift relation, *i.e.*, the edit distance between them is always two. Although differ in the measure (Hamming distance and edit distance), the Gray code and the de Bruijn code share the property of a constant distance between neighbors. However, we prefer to use the Gray code because it has the hierarchical structure favorable for visualization such that all 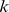-mers with a same prefix are included in a closed set, whereas the de Bruijn code does not have such a property.

Although the original Gray code is one-dimensional, the Gray code can be extended to two-dimensional (2D) space similar to CGR [12]. In the 2D Gray code, the 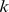-mers corresponding to two boxes adjacent in the vertical and horizontal directions differ from each other by only one nucleotide (Fig. 1(**b**)). Our biological knowledge suggests that similar sequences tend to have an identical or similar function, as exemplified by synonymous codons and iso-regulatory *cis*-elements. As a neighboring region always corresponds to a set of similar sequences, the 2D Gray code may be more useful than CGR that lacks such a property.

The 2D Gray code has a toric structure such that the top and bottom boundaries and the left and right boundaries are respectively connected to each other. This implies that the Gray code structure is closed on the surface of a torus. Unfortunately, however, it is difficult to realize a toric structure in the 3D space with an actual material, such as a paper craft.

**Figure 2:**
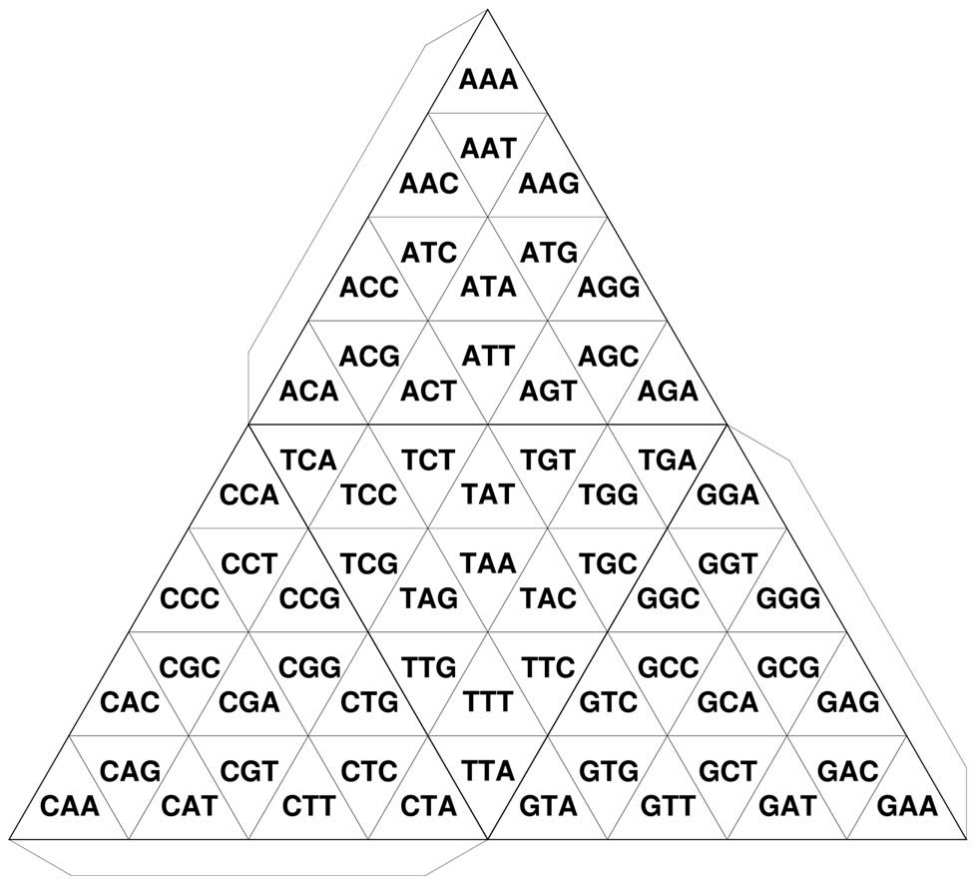
Transformation of TGC of trimers. Flaps indicate connecting boundaries in order to make a tetrahedron.

**Figure 3:**
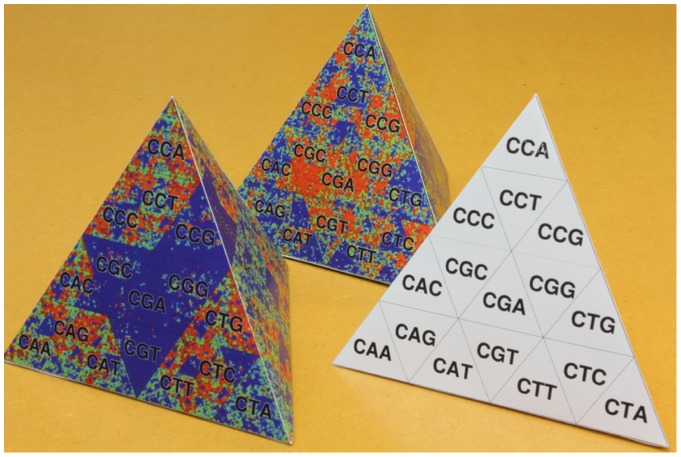
Paper crafts of TGC. Content information of human and honey bee genomes is depicted on the left and center paper crafts, respectively.

In this paper, we propose a tetrahedral Gray code (TGC) in which the Gray code is generated on tetrahedral surfaces. Whereas the basic unit of the 2D Gray code is rectangular, that of TGC is triangular (Fig. 2). In TGC, a 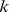-mer differs from each of its three neighbors by only one nucleotide. This relationship is valid even at the edges of the tetrahedron. We demonstrate here an algorithm and its implementation that enables us to draw the development of TGC on a paper with a conventional PC and a printer. As a tetrahedron can be easily constructed by paper craft (Fig. 3), the complete structure of the Gray code can be closely scrutinized in one's hands. Moreover, a set of tetrahedrons generated from the genomic sequences of various organisms can be displayed in a tree-structured object to visually demonstrate the evolutionary changes in genome contents along the tree of life.

## Methods

### Construction of tetrahedral Gray code (TGC)

**Figure 4:**
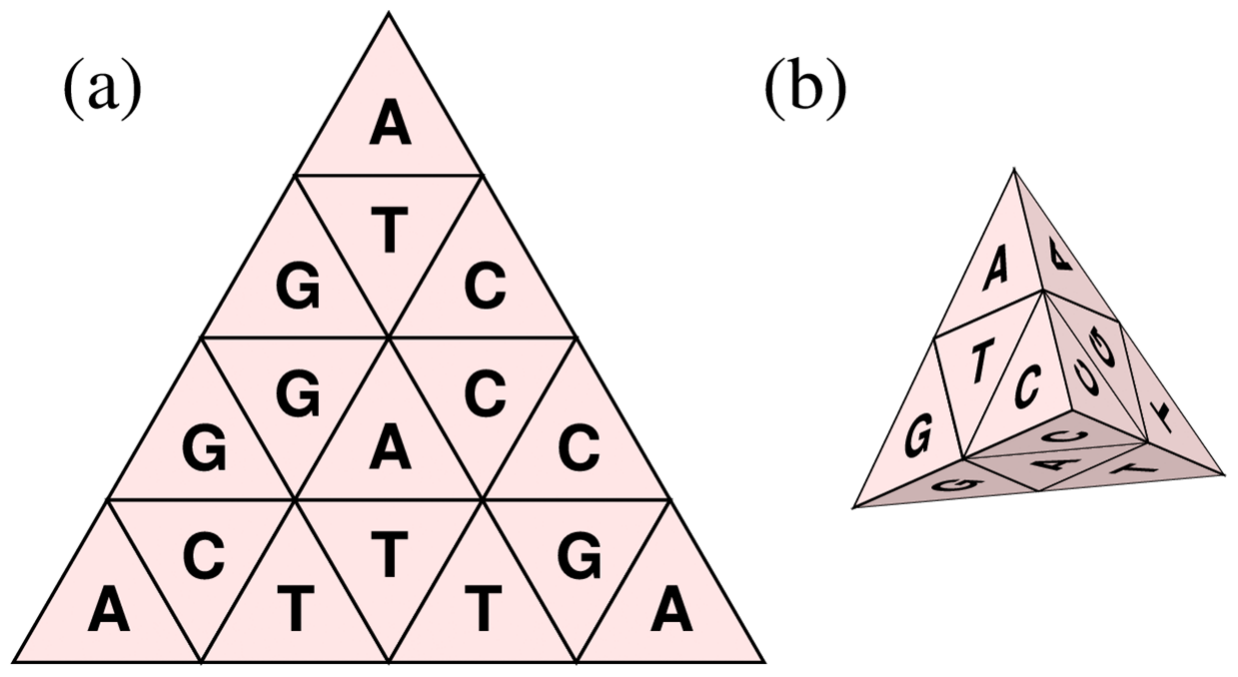
Generator represented by a development (a) and a tetrahedron (b).

We refer to a triangle corresponding to a 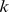-mer as a *cell*. We generate the 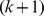-mer codes from a 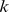-mer code by dividing the original cell into four smaller sub-cells corresponding to the four nucleotides, A, C, G, and T, which are appended to the original 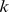-mer. The problem is how to assign the four nucleotides to the four sub-cells. In TGC, we use a specific *generator* tetrahedron to determine the assignment (Fig. 4).

**Figure 5:**
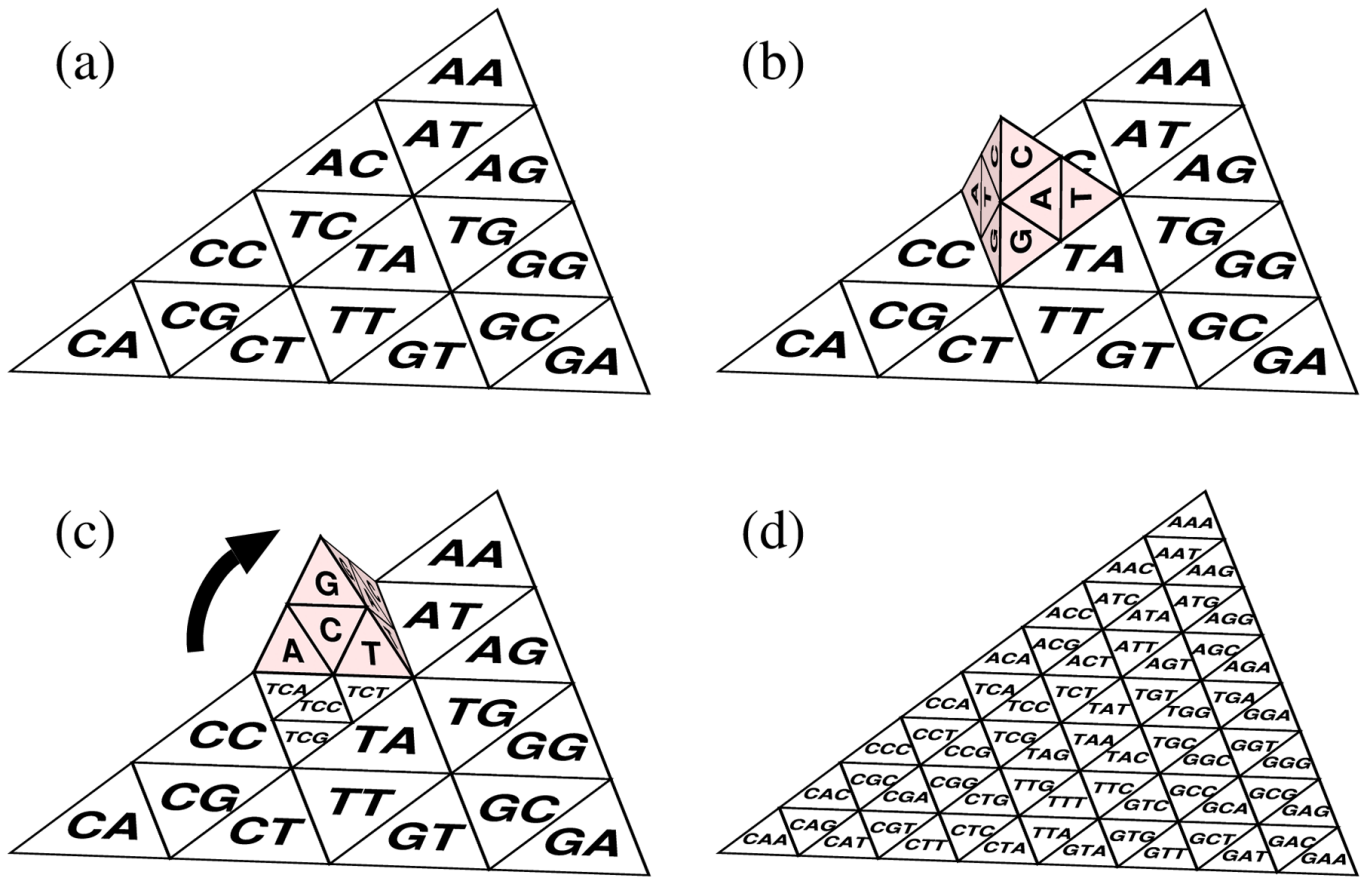
Procedure for formation of trimer code from dimer code. (**a**) The dimer code is assumed. (**b**) The generator is stamped on a cell. (**c**) The generator is rotated around an edge and stamped again. (**d**) Repeating the rotation and stamping for all cells yields the trimer code.

As illustrated in Fig. 4, each cell on a surface of the generator represents a unique nucleotide. An important feature of the generator is that the nucleotides corresponding to the two cells bordering each other on an edge are identical. To construct TGC, we start with the *monomer* tetrahedron whose four surfaces are labeled with the four kinds of nucleotides. Then, we recursively apply the following procedure up to the predefined depth, *i.e.*, the 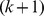-mer code is formed from the 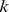-mer code by rotation and stamping of the generator, as shown in Fig. 5 (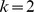 in this case). As a result of the stamping, the parental cell is divided into four sub-cells. By appending the nucleotides on the surface of the generator to the parental 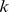-mer, we generate four unique 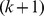-mers, which are then assigned to the corresponding sub-cells (Fig. 5(**c**)). All the cells in the original 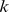-mer TGC are stamped by rotating the generator around each edge (Fig. 5(**d**)). Obviously, all possible 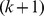-mers are generated by this inductive procedure and these 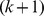-mers are unique. After the procedure reaches the predefined depth, we apply the genome information to TGC as described in the later subsection “Visualization of genome contents”.

We argue that this procedure produces TGC from the following observations. If two adjacent cells in the 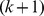-mer TGC are derived from the same parental (*i.e.*, 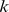-mer) cell, their sequences differ by only one nucleotide because the first 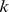-mers are identical while the last ones are unique because of the feature of the generator. On the other hand, if the two adjacent cells come from different parental cells, those parental cells must have been adjacent. Here, we assume that the parental cells satisfy the TGC conditions, *i.e.*, the sequences of the parental cells differ by only one nucleotide. The last nucleotides of those adjacent 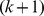-mers are identical because the nucleotides on both sides of an edge in the generator are identical. Therefore, the sequences of two adjacent cells differ by only one nucleotide even if those cells come from different parental cells. Consequently, the 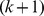-mer code is inductively a TGC as the monomer code is obviously a TGC.

The above consequence is dependent on the assumption that the generator can return to the first cell with the same surface and orientation after a certain number of rotations are applied to it. If this assumption were invalid, inconsistencies would occur in some cells during the procedure, *i.e.*, two or more different 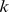-mers would be mapped in the same cell. The assumption of the recurrence of the generator is valid as proved in the next subsection.

### Recurrence of generator

Akiyama has shown that a tetrahedron can return to the first cell with the same surface and orientation after a certain number of rotations over a triangular lattice on a *plane* [13], [14]. However, movements of the generator on the plane and on the surfaces of the tetrahedron are not exactly the same because the tetrahedron is closed at its edges. A proof on the plane is therefore not directly applicable to that on the tetrahedron. In this regard, we have to show that the recurrence of the generator holds even when the generator moves over the edges of the tetrahedron.

**Figure 6:**
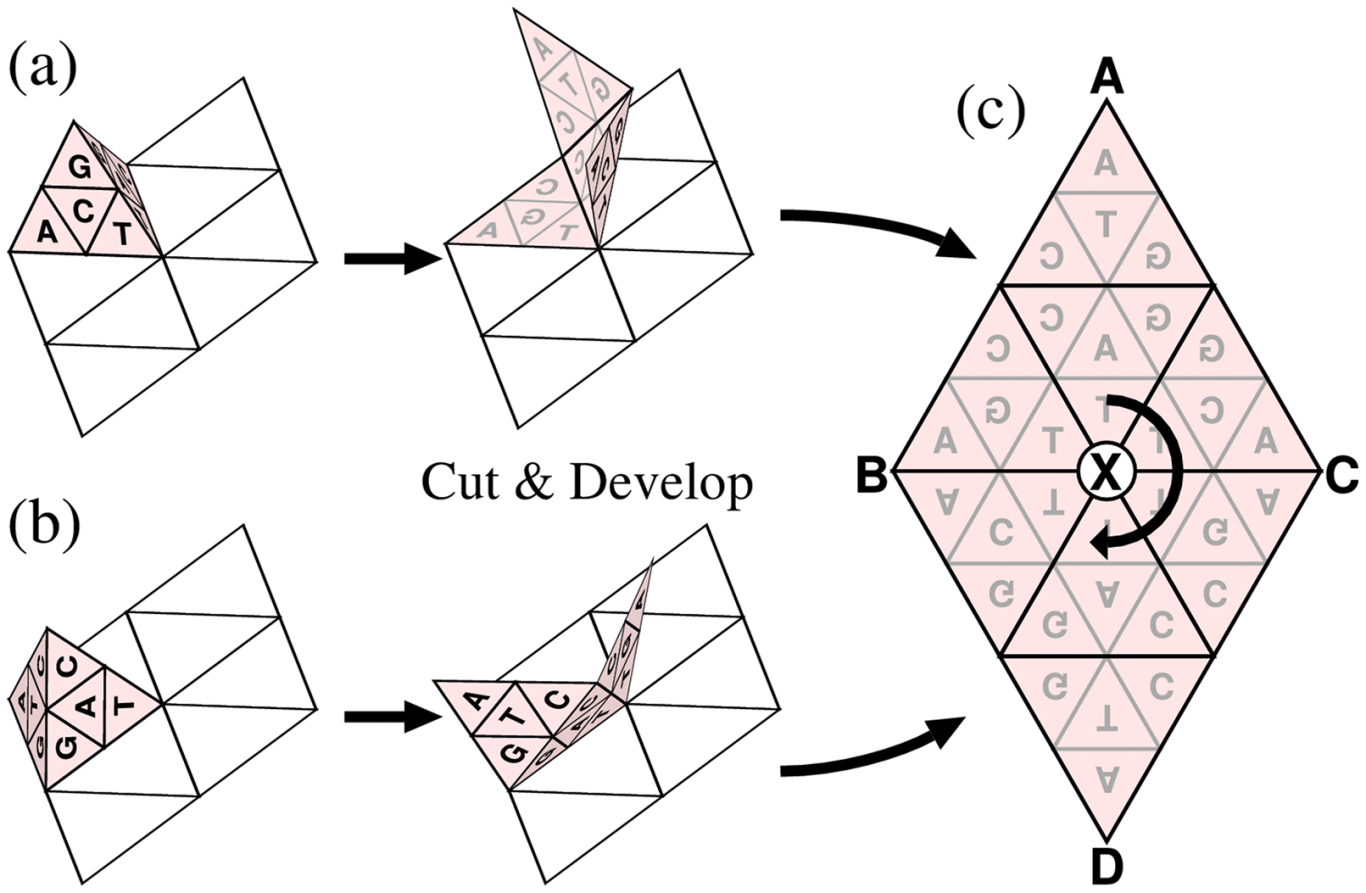
Equivalence between edge rotation of a tetrahedron and half turn of its development. (**a**) The tetrahedron before an edge rotation. (**b**) After an edge rotation of (**a**). (**c**) Triangles ABC and DCB are the developments of (**a**) and (**b**), respectively. These developments are related by the half turn around point X.

The key point is that the edge rotation of the generator should be equivalent to a half turn (180°) of the triangular development of the generator around each midpoint of its three boundaries. Consider two generators with the states before and after an edge rotation as shown in Fig. 6(**a**) and (**b**), respectively. These generators are developed by cutting open the same three edges including the rotation edge such that each development becomes a triangle. As a result, we obtain the two triangular developments ABC and DCB as shown in Fig. 6(**c**). These triangles including their internal patterns are identical to each other when the half turn around midpoint X of boundary BC is applied to them, implying the equivalence of the rotation of the generator around edge BC and this half turn. This relationship holds for the other two midpoints of boundaries AB and CA.

Similarly to the movement of a tetrahedron [13], [14], the recurrence of the triangle holds on the plane because the process of half turns generates a type of transformation group called *the plane crystallographic group* or *the wallpaper group* in group theory (Group p2 in our case) [15]. By the half turns, the development of the generator moves on the triangular lattice on which each triangle consists of four cells. The wallpaper group is defined as a set of such transformations and their compositions. As each half turn does not change the stamps as shown in the previous paragraph, its repeat (or composition) does not also change them. Therefore, all transformations belonging to the wallpaper group do not change the stamps.

We remark here that the invariance of stamps does not always imply the recurrence of the triangle on the plane, *i.e.*, it is only a necessary condition. Thus, we have to show that such a discordance does not occur in our case. In general, we can assume five transformations besides the identical transformation, such that a regular triangle is transformed into itself as a result of the composition of a certain number of transformations: two 120° rotations around the center and three reflections. However, these five transformations change the stamps and hence the assumption is inconsistent with the fact that a composition does not change the stamps. Therefore, only the identical transformation is allowable as the composition such that the triangle returns to the initial position after a certain number of half turns, proving the recurrence of the triangle on the plane [15].

**Figure 7:**
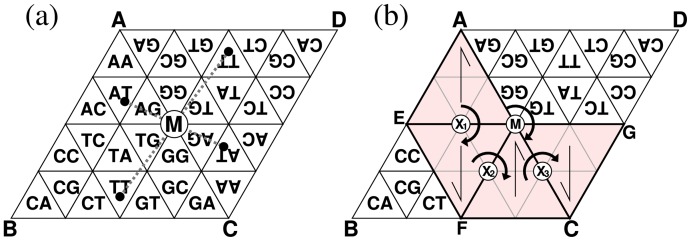
Development of a TGC (ABC) and its half turn (CDA) (a) and movements of the generator (b). (**a**) The positions of identical cells have point symmetry around M as shown by dotted lines. (**b**) The generator AEM successively moves to FME, MFC, and CGM by half turns at X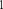, X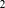, and X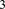, respectively. Each arrow inside the generator indicates its orientation. The initial triangle AEM and the last triangle CGM have point symmetry at M.

To emulate the movement of the generator on the surfaces of the tetrahedron, we consider the development of a TGC (triangle ABC) and one of its half turns (CDA) in Fig. 7(**a**). The movement of the generator over boundary AC corresponds to its movement over the edge of the tetrahedron. Segments AM and CM contact each other at the edge of the tetrahedron made from triangle ABC. The correspondence holds because segment AM (or CM) of triangle CDA is the transformed (turned) image of segment CM (or AM) of ABC, *i.e.*, these segments of the two developments contact each other in the same way as they contact at the edge of the tetrahedron. This correspondence holds for the other half turns of ABC. This fact implies that the recurrence of the generator on the tetrahedral surfaces is equivalent to the proposition that “a pair of identical cells in the original and transformed images has an identical stamp when the generator moves over the boundary between the two developments”. Note that the identical stamp implies the identical orientation of the generator as shown in the previous paragraph. Therefore, we only have to prove this proposition to complete the proof of the recurrence of the generator.

The movement of the generator over boundary AC is shown in Fig. 7(**b**). The three half turns around points X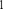, X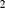, and X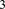 move the generator from the initial AEM to the last CGM positions. Triangles AEM and CGM have point symmetry around M. Moreover, the identical cells are also located symmetrically around M as shown in Fig. 7(**a**). Therefore, each pair of identical cells has an identical stamp. This property holds for longer 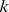-mers because the half turn around M belongs to the wallpaper group and hence all stamps are symmetric around M. Consequently, the above proposition is proved and then the proof for the recurrence of the generator on the tetrahedral surfaces is completed.

### Algorithm

**Figure 8:**
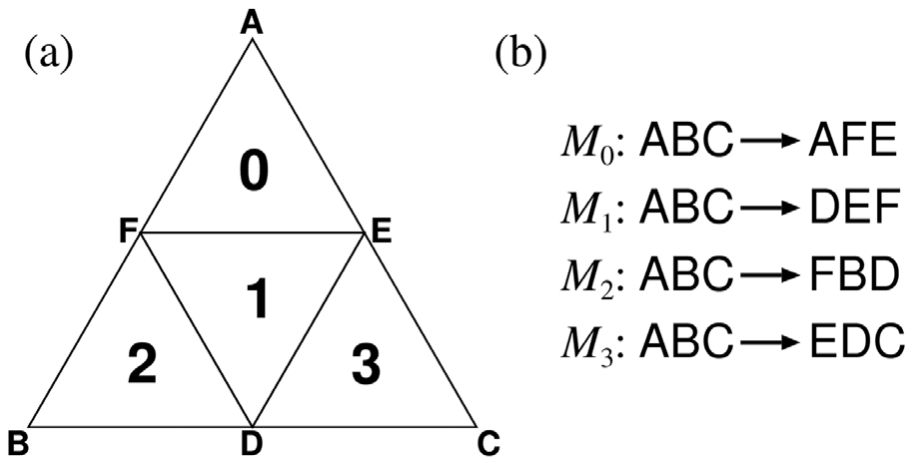
Relationship between addresses and cells (a) and affine transformations (b). Points A, B, and C are vertexes of a regular triangle and positioned at 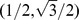, 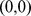, and 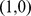, respectively. Points D, E, and F are the midpoints of the three boundaries, respectively. Each affine transformation moves triangle ABC into an inner triangle indicated in (**b**).

To draw TGC with a computer program, we first establish a relationship between a position of a triangular cell and a quaternary sequence that we call an *address*, 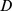, which is defined by:
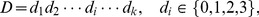
(1)where the size of 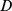 equals 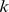 when we use 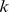-mer codes. The four digits assigned to each quaternary base 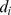 correspond to the four affine transformations defined by:
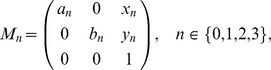
(2)where,



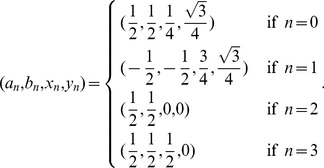
(3)Each affine transformation maps a regular triangle of unit length into an inner triangle as explained in Fig. 8. To transform a point 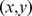, we use a vector 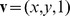 and a product 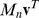, where 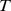 denotes the transposition.

As the product of these affine transformations, we obtain a transformation 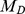 of the address 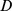:
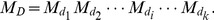
(4)


**Figure 9:**
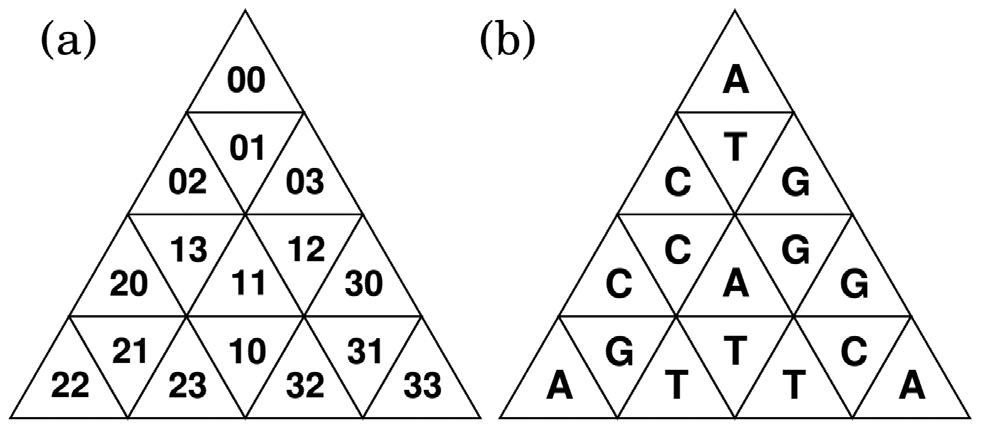
Address of length 2 (a) and generator (b). Note that (**b**) is a mirror image of the generator because this is a stamped image.

The transformation of the regular triangle ABC by 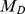 identifies the cell corresponding to the address 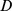. The relationship between the address of length 2 and the cell is exemplified in Fig. 9(**a**).

**Figure 10:**
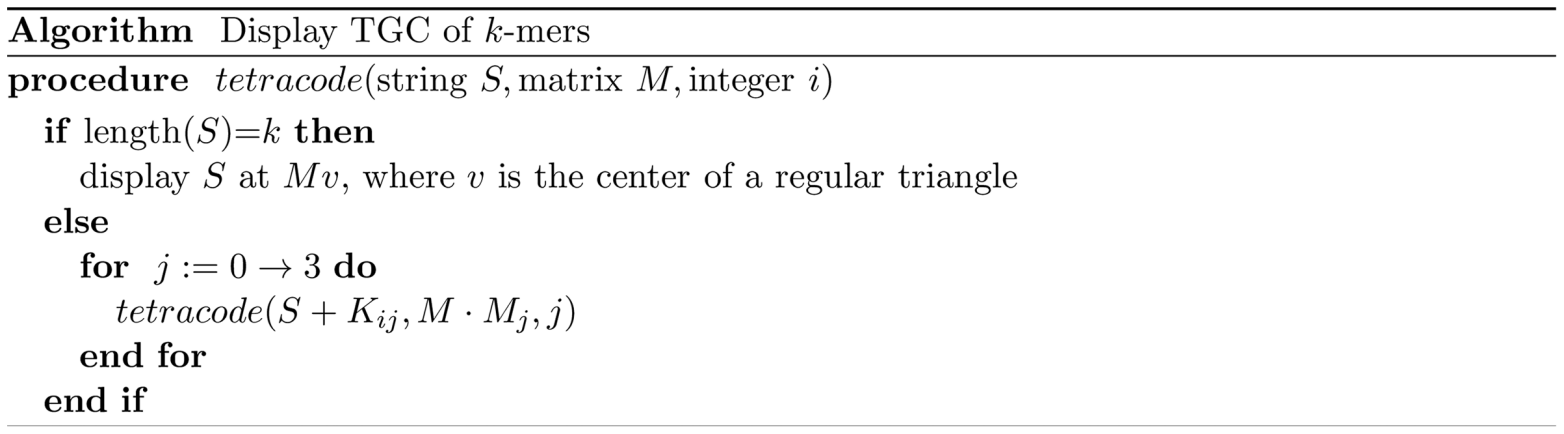
Algorithm for displaying TGC of 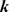-mers.

Each address 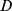 is associated with a 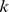-mer DNA sequence through the generator stamping rule. Fig. 9 shows an example of this association in the case of 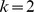, where each nucleotide in the generator specifies the second nucleotide of the dimer. The assignment of the first nucleotide is arbitrary and we here assign the first bases (0,1,2,3) into (A,T,C,G). Consequently, for example, the addresses (10,11,12,13) are assigned into (TT,TA,TG,TC) and (20,21,22,23), into (CC,CG,CA,CT). Note that a base in the address does not directly represent the corresponding nucleotide but the assignments of the second bases depend on the first base of the address. This assignment rule is recursively applicable to a pair of 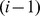-th and 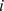-th bases, and the address-to-sequence transformation 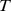 can be expressed as:
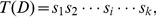
(5)

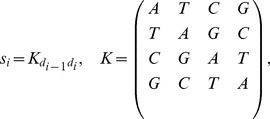
(6)where 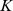 is the relation matrix defined by the generator, 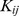 denotes an element of 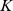, and 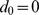 is arbitrarily chosen to assign the first bases. The algorithm for the construction of TGC is summarized in the pseudocode (Fig. 10). All 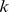-mers are displayed by calling 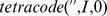, where 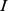 is the 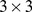 identity matrix.

### Visualization of genome contents

We use TGC to visualize genome information represented by the 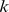-mer frequency 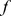, which is defined by:
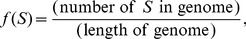
(7)where 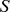 denotes a 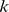-mer, 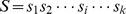, 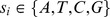. For sequence analysis, 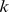-mer frequencies relative to some *background* frequencies are often more useful than the raw values. For example, we can adopt the zeroth-order Markov model as the background frequencies 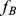:
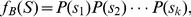
(8)where 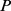 is the probability of occurrence of a nucleotide in the genome. Other examples of background frequencies are a higher-order Markov model for emphasis of longer-range sequence characteristics and 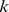-mer frequencies of another genome for comparative genomics.

**Figure 11:**
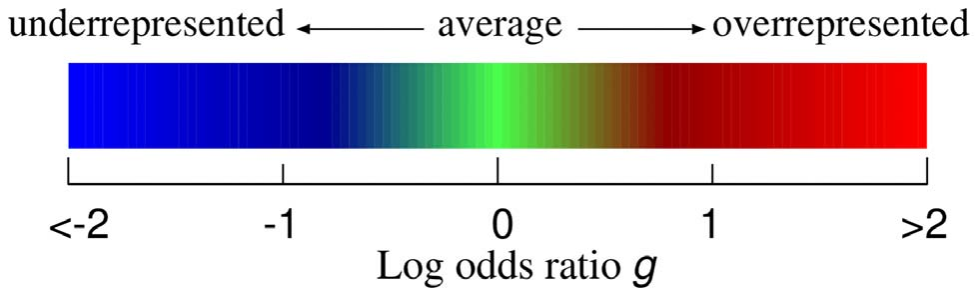
Color coordinate of log odds ratio 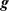.

To demonstrate the contrast between the observed and background 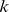-mer frequencies, we use the log odds ratio 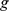 defined by:
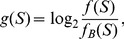
(9)which is color-encoded (Fig. 11) and depicted on TGC.

## Results and Discussion

### Educational use of TGC

**Figure 12:**
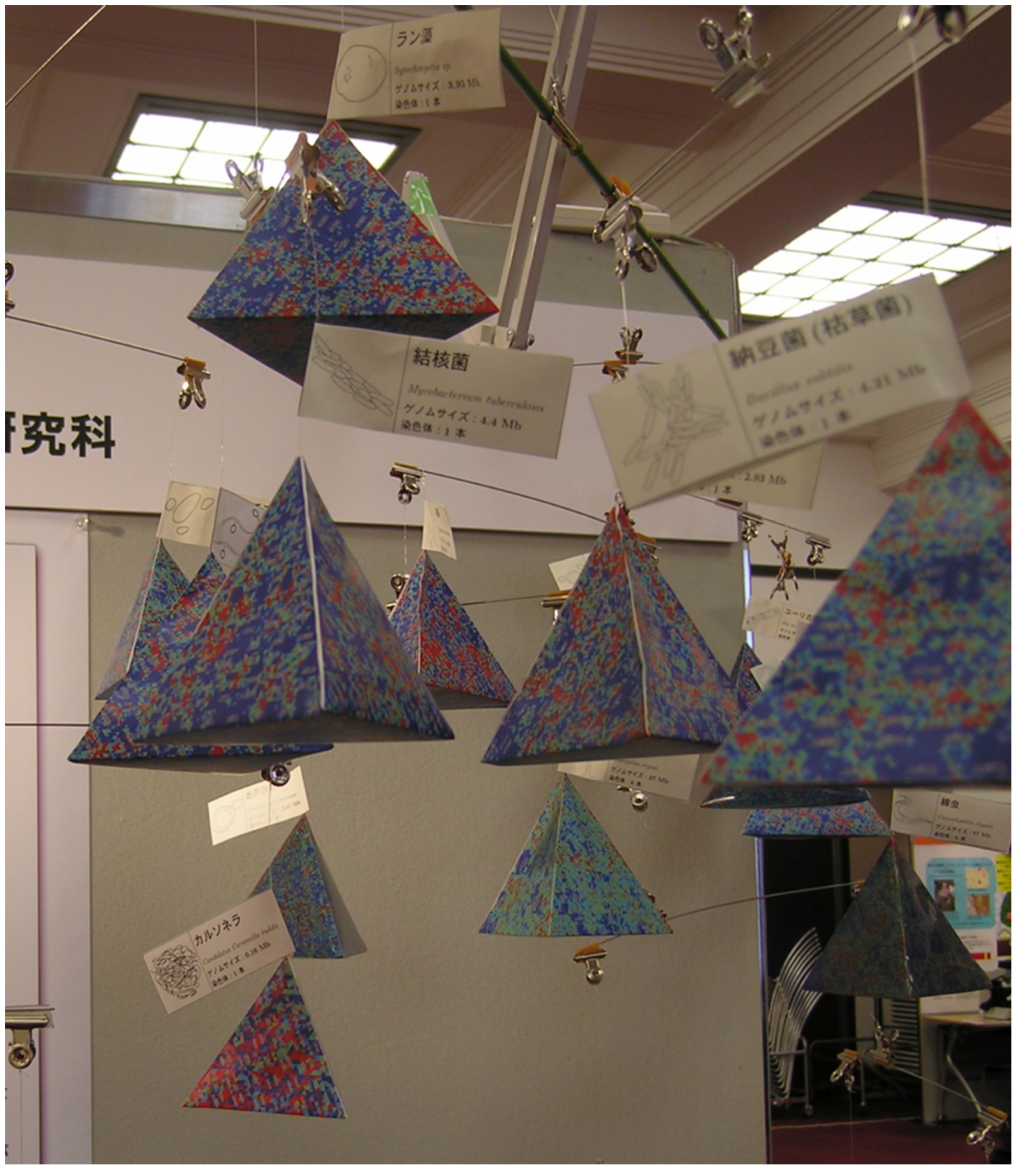
Exhibition of TGCs in a science outreach event. The mobile sculpture is composed along the tree of life.

In a science outreach event, we used TGC to exhibit the genomic landscape along the tree of life. The genomic landscape is displayed in a mobile sculpture that is arranged to accord with a phylogenetic tree composed of 34 organisms (Fig. 12 and S1). Specifically, in the mobile sculpture, a fulcrum corresponds to a branch point of organisms and an object dangling from a horizontal bar by a rod corresponds to a TGC expressing the genome information of a specific organism.

In the mobile sculpture, we can observe evolutionary conservation and variation among neighboring organisms at a glance. For example, organisms in vertebrates have similar patterns in TGC, whereas those in insects are more diversified as we discuss in the following subsections in detail. Thus, this mobile sculpture can give us a concise insight into comparative genomics.

For calculation of a TGC of the complete human genome (total size of 2.95 Gbp), for example, the calculation time was 28 s (linearly dependent on genome size) and the memory usage was 17 MB on a normal PC. Thus, the application of TGC is sufficiently feasible for creating a large mobile sculpture. Furthermore, we provide an auxiliary tool named GENOREP to select an appropriate number of representatives from a potentially huge set of genomic sequences as described in Text S1.

### Analytical use of TGC

#### Genome information and CpG methylation

**Figure 13:**
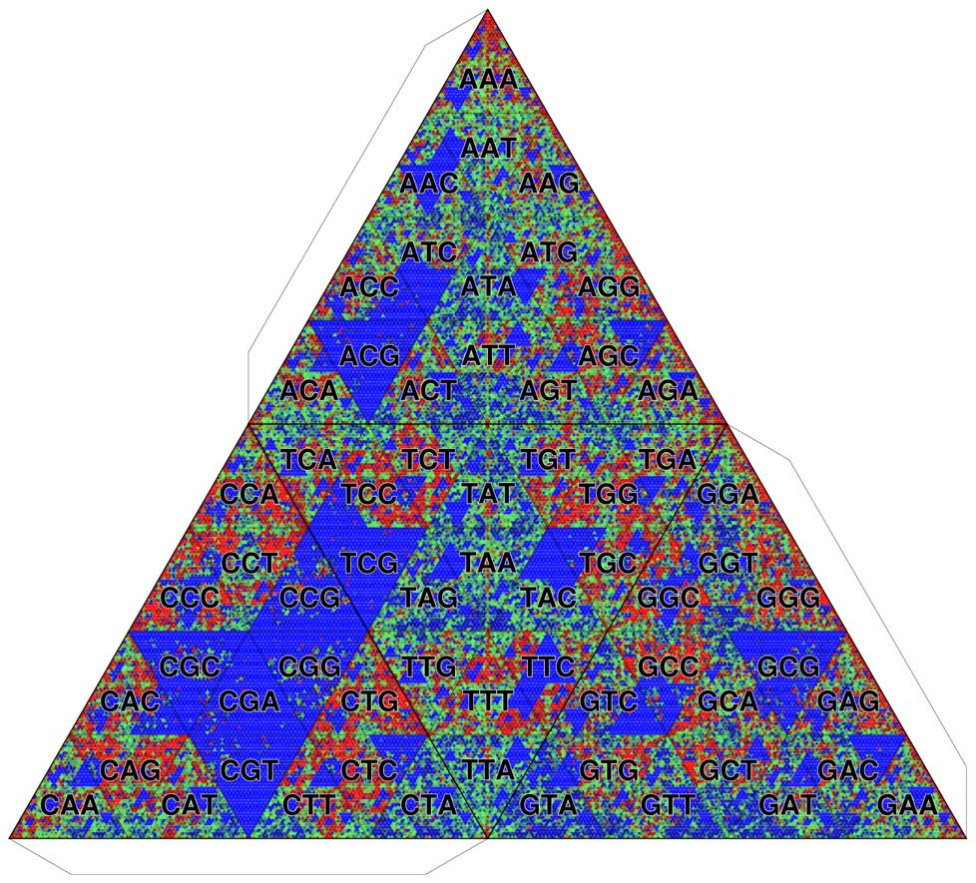
TGC of human genome (*Homo sapiens*). Octamer frequencies are depicted. The background frequency is determined by the zeroth-order Markov model.

Figure 13 shows the TGC of the human genome. For this and other examples, we use the frequency distribution of octamers (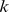 = 8) for color visualization. Since the number of the cells is huge (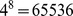 for octamers), we indicate only the first three letters common to a closed set of octamers. This display is feasible because the Gray code has the hierarchical structure as we mentioned in Introduction. The background frequency is determined by the zeroth-order Markov model that is constructed from the given genome itself. The most remarkable feature perceivable in Fig. 13 is the depletion of 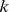-mers having CpG dinucleotides, which is demonstrated by the large blue area around the prefix CG. The CpG depletion is caused by the methylation of the cytosine of CpG [16]. The spontaneous deamination of methyl-cytosine causes mutations to thymine, which are difficult to repair as thymine is a normal DNA component. As the methylation of CpG is adopted as the functional regulator in vertebrates, the characteristics of CpG depletion are ubiquitously observed in vertebrate genomes (Fig. S2–S5).

**Figure 14:**
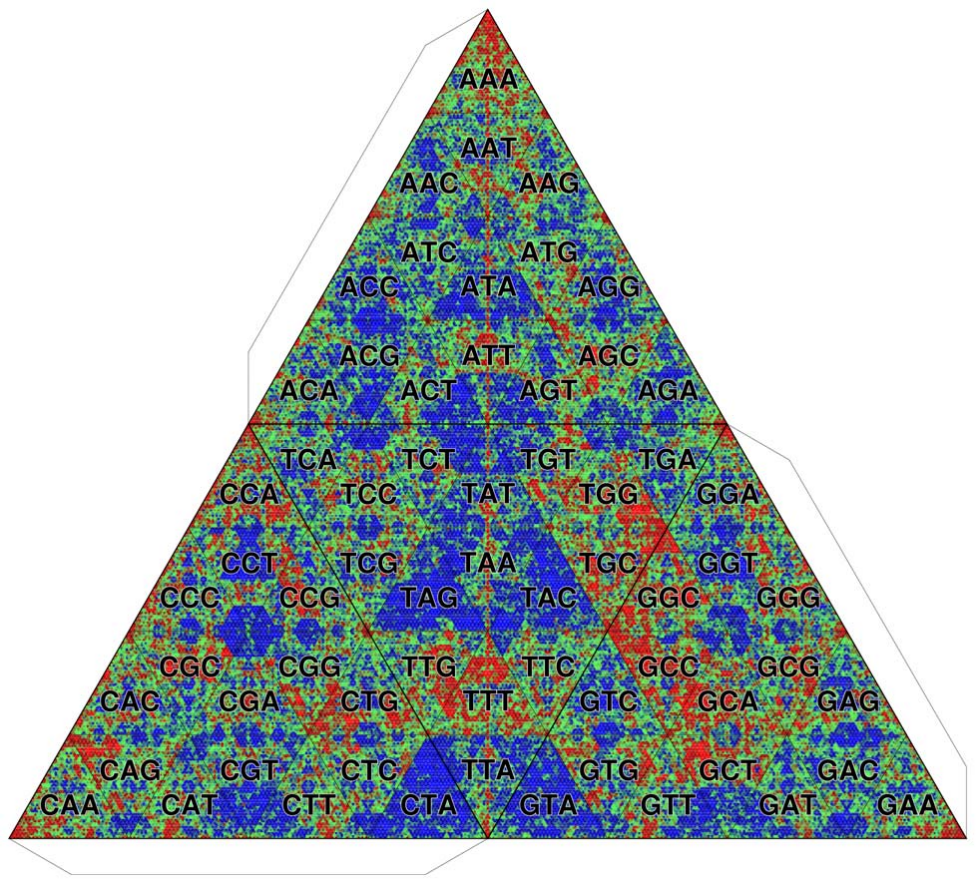
TGC of fruit fly genome (*Drosophila melanogaster*). Octamer frequencies are depicted. The background frequency is determined by the zeroth-order Markov model.

**Figure 15:**
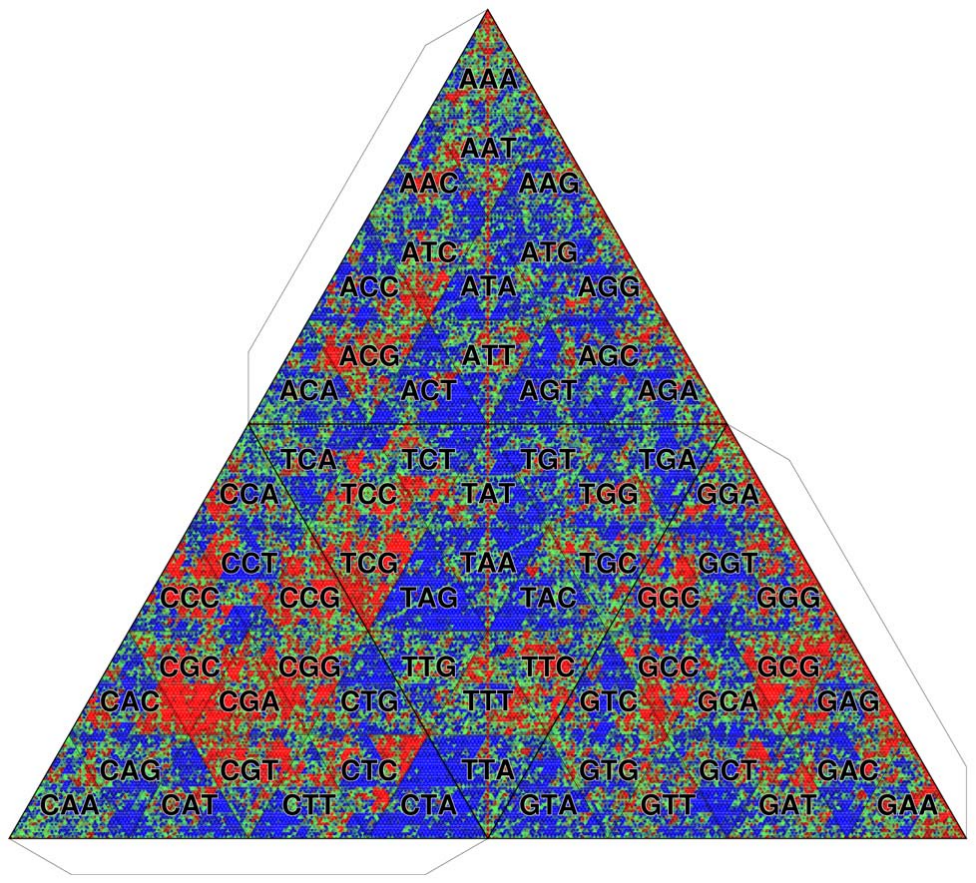
TGC of honey bee genome (*Apis mellifera*). Octamer frequencies are depicted. The background frequency is determined by the zeroth-order Markov model.

In invertebrates, such as insects, the patterns of methylation are much more diversified. In accordance with the observation that the methylation of the fruit fly (*Drosophila melanogaster*) genome is restricted to the early stages of embryonic development [17], appreciable CpG depletion is not observed (Fig. 14). By contrast, the CpG methylation in the honey bee (*Apis mellifera*) genome contributes to important developmental determinations to become a queen or a worker [18]. Unexpectedly, however, the CpG-containing 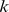-mers are overrepresented (Fig. 15) in contrast to the observations of vertebrate genomes. To solve this discrepancy, we analyze the methylation of the *A. mellifera* genome in more detail.

#### Methylation of *A. mellifera* genome

The methylation status of the *A. mellifera* genome was obtained from the results of bisulfite sequencing of queen brain genome [19] (accession number: SRA012457) by means of the same method as that explained in the original paper [19].

It is known that the methylation sites are specifically located at exons [19] and CpG methylation of them is used to control the activities of the genes [20]. Then, we classify a gene into two categories, methylated and unmethylated, by the criterion whether the gene has at least one methylated exon or not. The CpG observation/expectation (o/e) ratios of the *A. mellifera* genes (*i.e.*, 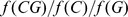) have a bimodal distribution [20] and the methylated genes are categorized into the low CpG class [19]. Indeed, the average CpG (o/e) ratio of the methylated genes is 0.632 whereas that of the unmethylated genes is 1.13. This result indicates that the CpG depletion actually occurs in the methylated genes.

Because of their larger sizes, intergenic regions have a greater contribution than intragenic regions to the characteristics of the whole genome. Then, we extract two types of intergenic regions, those between methylated genes (IG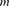) and those between unmethylated genes (IG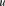). Although the discrimination between IG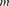 and IG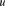 is made by the methylation states of its adjacent genes but not by those of IG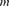 and IG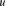 themselves, IG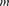 has significantly more methylation sites than IG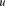 (Table S1). In addition, the length of IG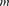 (average 2,880 bp and total 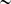
9 Mbp) is one order of magnitude less than that of IG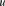 (19,133 bp and 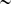
39 Mbp) (Fig. S6). Therefore, IG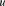 is mainly responsible for the CpG overrepresentation of the *A. mellifera* genome.

**Figure 16:**
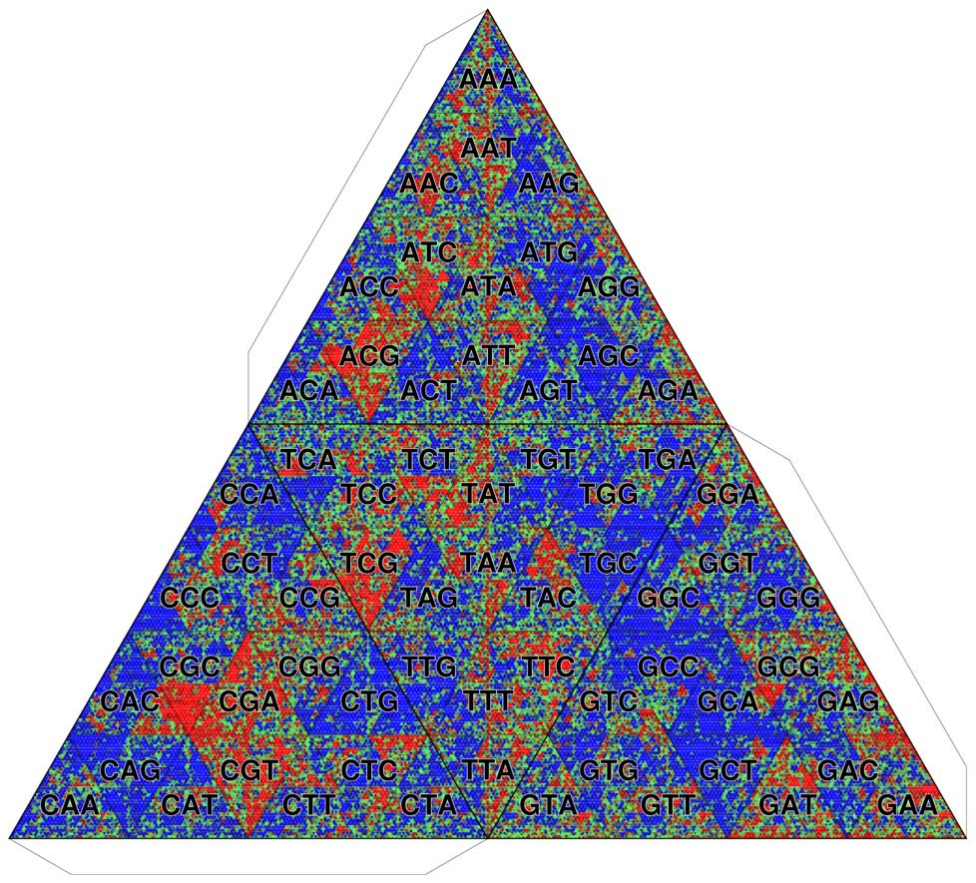
Comparative visualization of IG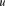 of *A. mellifera* genome with intergenic regions of *D. melanogaster* genome. Octamer frequencies are depicted. The background frequency is determined by *D. melanogaster* genome.

It is still unclear whether the CpG overrepresentation in IG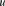 is simply explained by the lack of methylation. To answer this question, we compare IG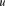 with the intergenic regions of the *D. melanogaster* genome which is not methylated at the adult stage [17]. TGC is critical for such a comparative analysis, in which we use the intergenic sequences of *D. melanogaster* as the background frequency 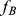 in Equation (9). The result shows that the CpG-containing 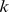-mers are more overrepresented in the *A. mellifera* IG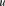 than in the intergenic regions of *D. melanogaster* (Fig. 16), suggesting that the CpGs in *A. mellifera* IG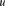 are maintained more actively than the passive effect of unmethylation.

**Figure 17:**
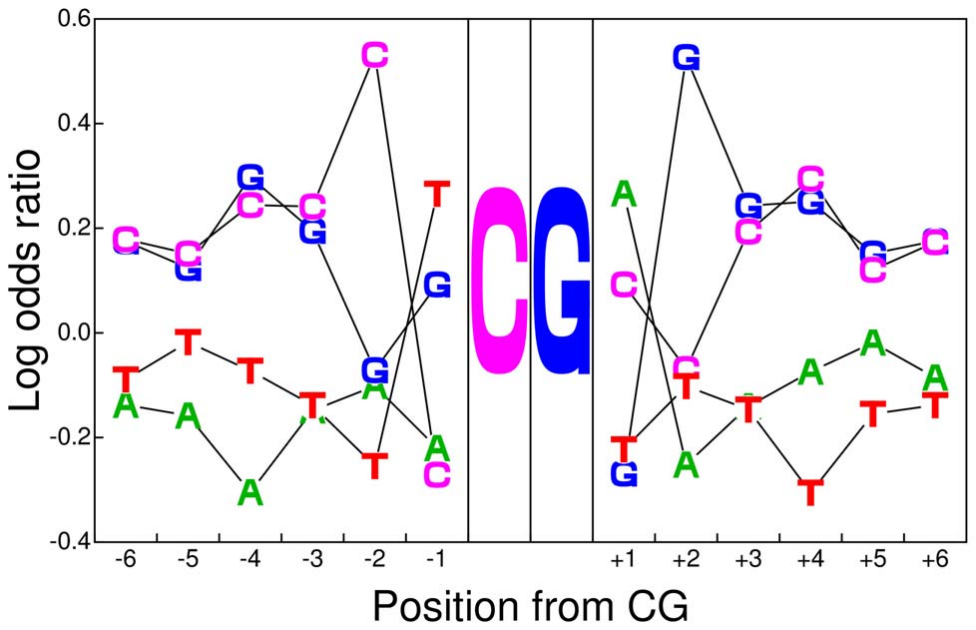
Motif around CpG in IG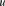 regions.

It is notable that not all but only particular CpG-containing sequences, *e.g.*, CGA, CGT, ACG, TCG, and CGCG, are overrepresented (Fig. 16). This result suggests the existence of motifs around CpG. To find a potential motif(s) around CpG in IG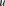, we plot the weight matrix, *i.e.*, the log odds ratio 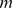 of the conditional probability:
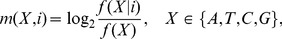
(10)where 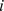 is the position from CG and 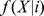 indicates the conditional frequency of nucleotide 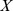 at position 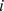. As shown in Fig. 17, the most prominent motif is CTCGAG and the second most prominent motif is CGCGCG.

The consensus sequence CTCGAG coincides with the recognition site of restriction enzyme *Xho*I. This enzyme is known to recognize unmethylated CpG, suggesting that the consensus sequence may be the binding target of a protein that has DNA binding ability and competes with methyltransferase. By contrast, the consensus sequence CGCGCG can originate from tandem repeats of CpG. Because it is known that tandem repeats tend to be unmethylated in the *A. mellifera* genome [21], this motif may be involved in the mechanisms preventing DNA in tandem repeats from methylation. These motifs are overrepresented in IG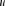 in comparison with IG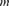 and these characteristics are also conserved in related species (Fig. S7 and S8). Consequently, CTCGAG and CGCGCG are considered to be functional motifs specific to the unmethylated regions and the reason for the CpG overrepresentation is that these consensus sequences having CpG are significantly conserved in IG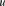 that occupies most of the *A. mellifera* genome.

The heterogeneity of the GC content within the *A. mellifera* genome has been studied by Kent *et al.* [22] in association with the higher recombination rate in GC-rich regions; they showed that the mutation rate of A/T to G/C is higher than the reverse rate because of the higher recombination rate in GC-rich regions and hence the regions are maintained in GC-rich states. Since the CpG (o/e) ratio and the GC content are statistically independent of each other, the result of Kent *et al.* does not directly explain the CpG overrepresentation in *A. mellifera*. However, the existence of the GC-rich motifs we found is consistent with their result because the motifs should be conserved and hence the mutation rate of G/C to A/T should be suppressed in the motifs.

## Conclusions

We have proposed the tetrahedral Gray code (TGC) to visually represent the genome information of various organisms. The mobile sculpture of TGC is informative for comparative genomics. Indeed, we got the idea of the specificity of the *A. mellifera* genome by observing this mobile sculpture. For a single genome, the TGC condition is useful for finding motif-like structures observed as a contiguous region with high frequencies. The boundary of such a region is also important. As 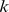-mers bordering each other on the boundary differ by only one nucleotide, the drastic change of their frequencies ensures the importance of this divergent nucleotide. This contiguous property helps us to better understand the observed characteristics compared with CGR in which neighboring 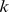-mers can be completely different.

The structure of TGC is clarified by making its paper craft. In order to make a paper craft of given genomes, the online and stand-alone versions of the application, named Padog, are available at our website:

http://www.genome.ist.i.kyoto-u.ac.jp/~ichinose/padog/.

## Supporting Information

Figure S1 **Exhibition of TGCs of 34 organisms in a science outreach event.** The mobile sculpture is composed along the tree of life.
(TIFF)

Figure S2 **TGC of mouse genome (** ***Mus musculus*** **).** Octamer frequencies are depicted. The background frequency is determined by the zeroth-order Markov model.
(TIFF)

Figure S3 **TGC of chicken genome (** ***Gallus gallus*** **).** Octamer frequencies are depicted. The background frequency is determined by the zeroth-order Markov model.
(TIFF)

Figure S4 **TGC of frog genome (** ***Xenopus tropicalis*** **).** Octamer frequencies are depicted. The background frequency is determined by the zeroth-order Markov model.
(TIFF)

Figure S5 **TGC of zebrafish genome (** ***Danio rerio*** **).** Octamer frequencies are depicted. The background frequency is determined by the zeroth-order Markov model.
(TIFF)

Figure S6
**Distributions of lengths of intergenic regions IG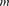 and IG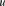.**
(TIFF)

Figure S7
**Normalized motif frequencies of CTCGAG for seven insects.** (A) honey bee, (B) dwarf honey bee, (C) buff-tailed bumblebee, (D) jewel wasp, (E) red imported fire ant, (F) silkworm, and (G) pea aphid. The asterisks imply that the motif is significantly enriched in IG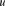 (significance level: 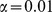).(TIFF)

Figure S8
**Normalized motif frequencies of CGCGCG for seven insects.** (A) honey bee, (B) dwarf honey bee, (C) buff-tailed bumblebee, (D) jewel wasp, (E) red imported fire ant, (F) silkworm, and (G) pea aphid. The asterisks imply that the motif is significantly enriched in IG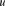 (significance level: 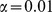).(TIFF)

Table S1
**Number of methylated or unmethylated CpG in IG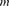 and IG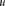.**
(TIFF)

Text S1 **Details of supporting information.**
(PDF)

## References

[1] Jeffrey HJ (1990) Chaos game representation of gene structure. Nucleic Acids Res., 18(8), 2163–2170. 10.1093/nar/18.8.2163 PMC330698 2336393

[2] Basu S, Pan P, Dutta C, Das J (1997) Chaos game representation of proteins, J. of Mol. Graphics and Modelling, 15, 279–289. 10.1016/s1093-3263(97)00106-x 9640559

[3] Almeida JS, Vinga S (2002) Universal sequence map (USM) of arbitrary discrete sequences. BMC Bioinfo. 3: 6. 10.1186/1471-2105-3-6 PMC90187 11895567

[4] Deschavanne PJ, Giron A, Vilain J, Fagot G, Fertil B (1999) Genomic signature: Characterization and classification of species assessed by chaos game representation of sequences. Mol. Biol. Evol., 16(10), 1391–1399. 10.1093/oxfordjournals.molbev.a026048 10563018

[5] Hao B-L, Lee HC, Zhang S-Y (2000) Fractals related to long DNA sequences and complete genomes, Chaos, Solitons and Fractals, 11, 825–836.

[6] Gutiérrez JM, Rodríguez MA, Abramson G (2001) Multifractal analysis of DNA sequences using a novel chaos-game representation. Physica A, 300, 271–284.

[7] Almeida JS, Carriço JA, Maretzek A, Noble PA, Fletcher M (2001) Analysis of genomic sequences by Chaos Game Representation. Bioinformatics, 17(5), 429–437. 10.1093/bioinformatics/17.5.429 11331237

[8] Gray F (1947) Pulse code communication. U.S. Patent 2632058.

[9] Er MC (1984) On generating the 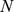 -ary reflected Gray codes, IEEE Trans. on Comp., C-33 (8), 739–741.

[10] Ichinose N, Yada T, Gotoh O (2012) Large-scale motif discovery using DNA Gray code and equiprobable oligomers. Bioinformatics, 28(1), 25–31. 10.1093/bioinformatics/btr606 PMC3244767 22057160

[11] de Bruijn NG (1946) A Combinatorial Problem. Koninklijke Nederlandse Akademie v. Wetenschappen, 49, 758–764.

[12] Feldman W, Pevzner P (1994) Gray code masks for sequencing by hybridization. Genomics, 23, 233–235. 10.1006/geno.1994.1482 7829077

[13] Akiyama J, Hirata K, Kobayashi M, Nakamura G (2006) Convex developments of a regular tetrahedron. Comp. Geometry, 34, 2–10.

[14] Akiyama J (2007) Tile-makers and semi-tile-makers. Amer. Math. Mon., 114(7), 602–609.

[15] Duzhin SV, Chebotarevskii BD (2004) Transformation groups for beginners, AMS, Student Mathematical Library 25.

[16] Cooper DN, Krawczak M (1989) Cytosine methylation and the fate of CpG dinucleotides in vertebrate genomes. Hum. Genet., 83(2), 181–188. 10.1007/BF00286715 2777259

[17] Lyko F, Ramsahoye BH, Jaenisch R (2000) DNA methylation in *Drosophila melanogaster*. Nature, 408, 538–540. 10.1038/35046205 11117732

[18] Kucharski R, Maleszka J, Foret S, Maleszka R (2008) Nutritional Control of Reproductive Status in Honeybees via DNA Methylation. Science, 319(5871), 1827–1830. 10.1126/science.1153069 18339900

[19] Lyko F, Foret S, Kucharski R, Wolf S, Falckenhayn C, et al.. (2010) The honey bee epigenomes: Differential methylation of brain DNA in queens and workers. PLoS Biol., 8(11), e1000506. 10.1371/journal.pbio.1000506 PMC2970541 21072239

[20] Foret S, Kucharski R, Pittelkow Y, Lockett GA, Maleszka R (2009) Epigenetic regulation of the honey bee transcriptome: unravelling the nature of methylated genes. BMC Genomics 10: 472. 19828049 10.1186/1471-2164-10-472 PMC2768749

[21] Feng S, Cokus S, Zhang X, Chen PY, Bostick M, et al.. (2010) Conservation and divergence of methylation patterning in plants and animals. PNAS, 107(19), 8689–8694. 10.1073/pnas.1002720107 PMC2889301 20395551

[22] Kent CF, Minaei S, Harpur BA, Zayed A (2012) Recombination is associated with the evolution of genome structure and worker behavior in honey bees, PNAS, 109(44), 18012–18017. 10.1073/pnas.1208094109 PMC3497793 23071321

