# Supplementary figures and images for: Tetrahedral Gray Code for Visualization of Genome Information
Source: PLoS One. 2014 Jan 27;9(1):e86133. doi: 10.1371/journal.pone.0086133 (PMC3903499; doi:10.1371/journal.pone.0086133)

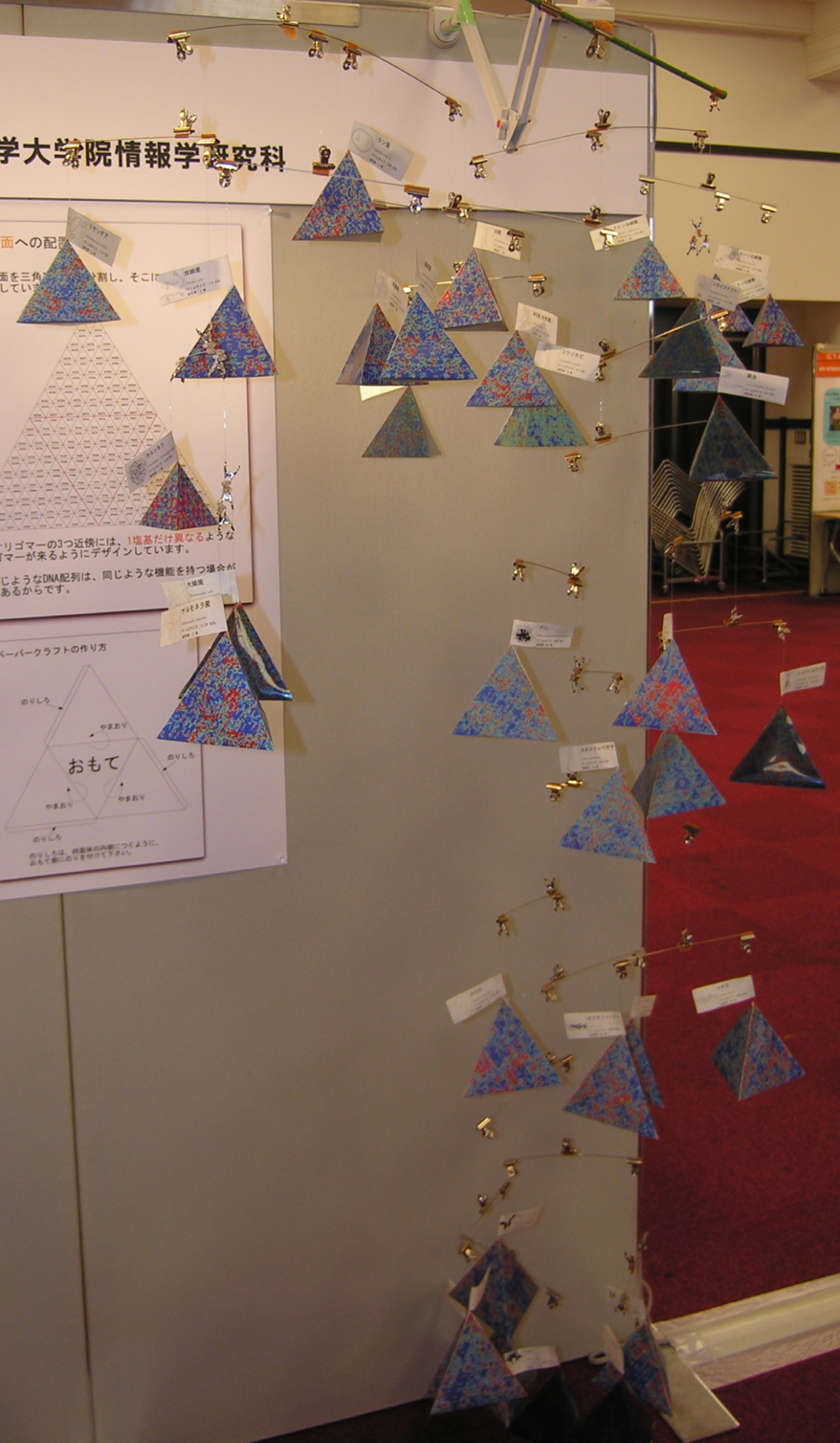

Supplement: Figure S1 — Exhibition of TGCs of 34 organisms in a science outreach event. The mobile sculpture is composed along the tree of life. (TIFF) [file pone.0086133.s001.tiff]

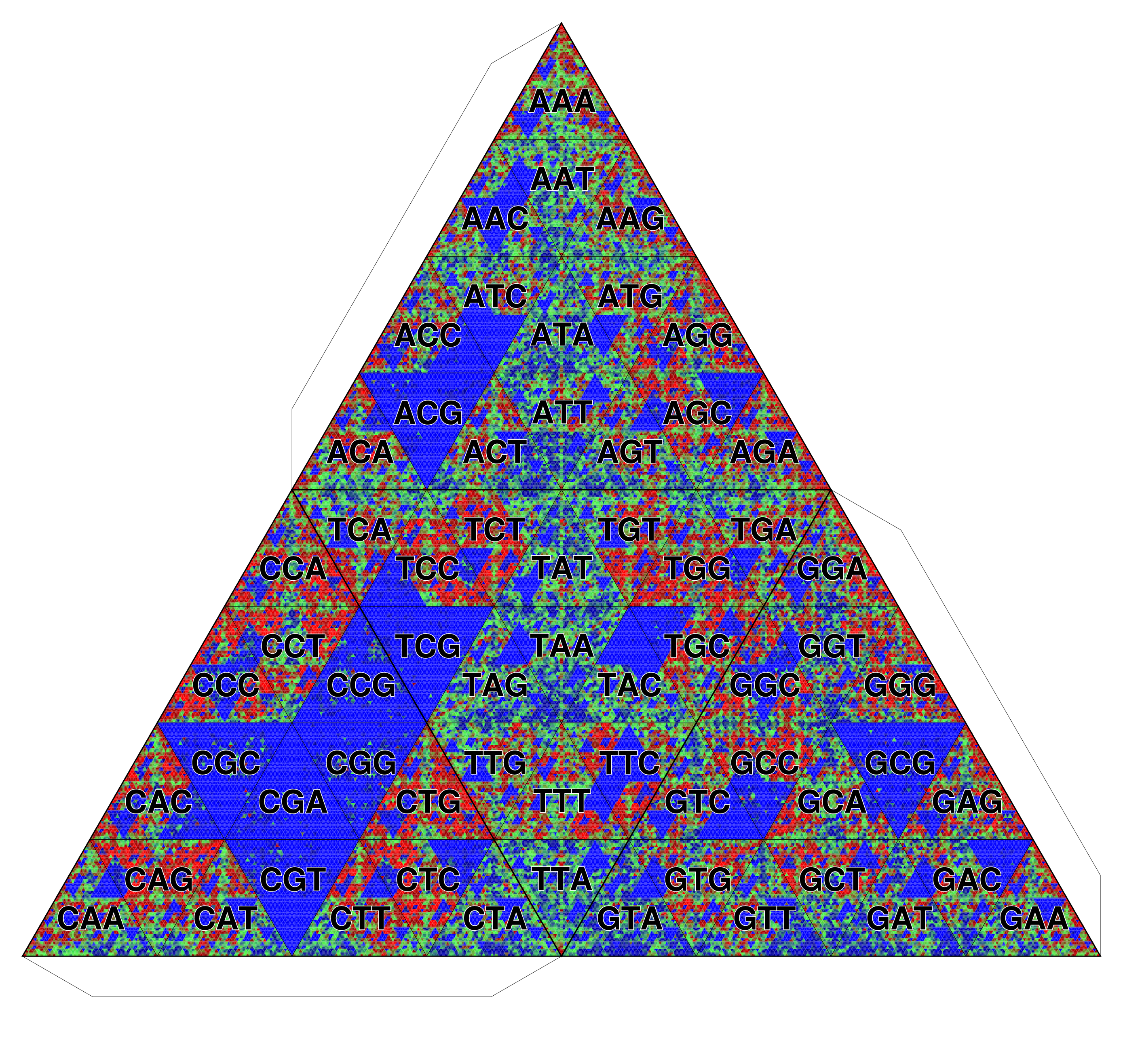

Supplement: Figure S2 — TGC of mouse genome ( Mus musculus ). Octamer frequencies are depicted. The background frequency is determined by the zeroth-order Markov model. (TIFF) [file pone.0086133.s002.tiff]

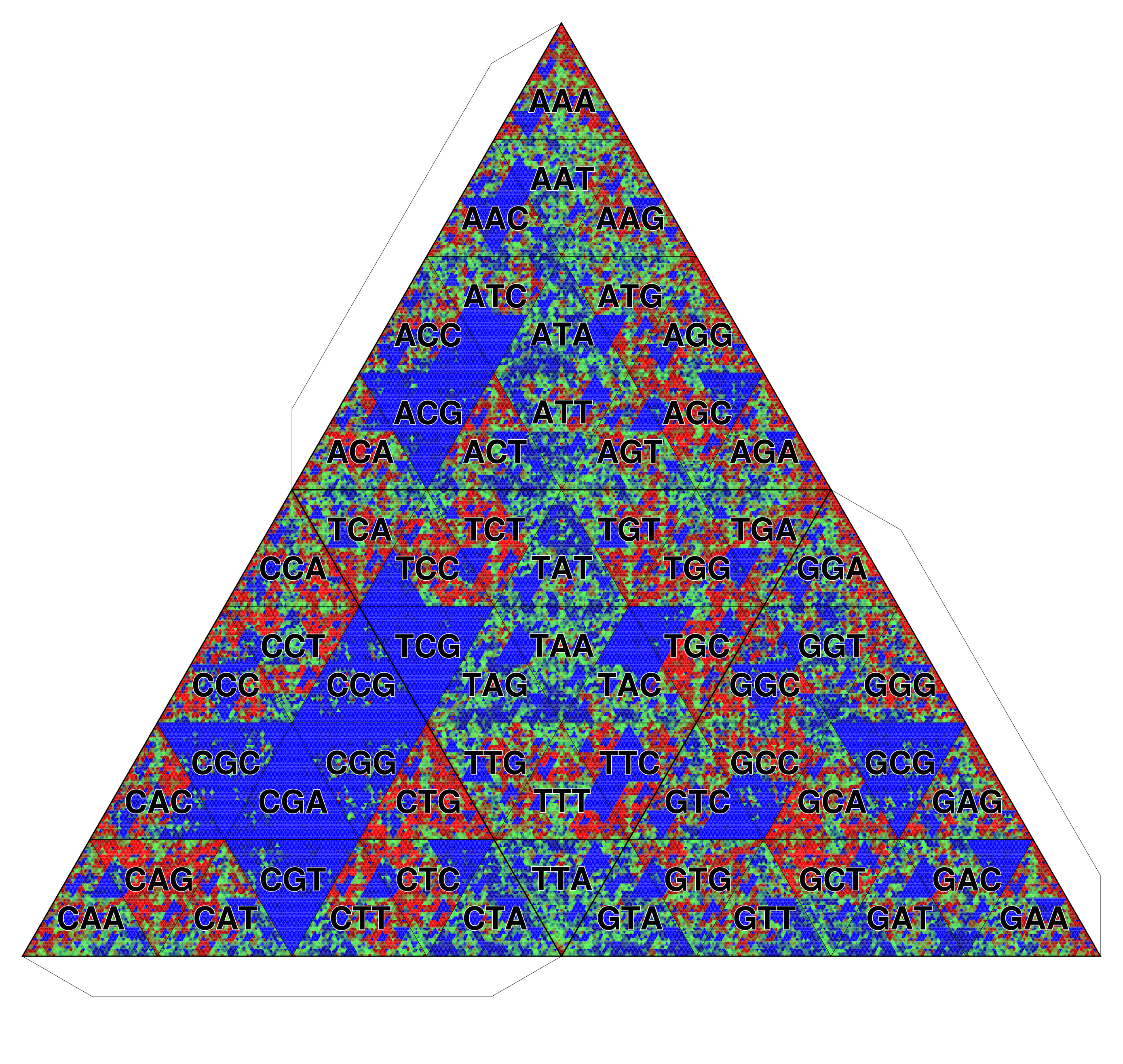

Supplement: Figure S3 — TGC of chicken genome ( Gallus gallus ). Octamer frequencies are depicted. The background frequency is determined by the zeroth-order Markov model. (TIFF) [file pone.0086133.s003.tiff]

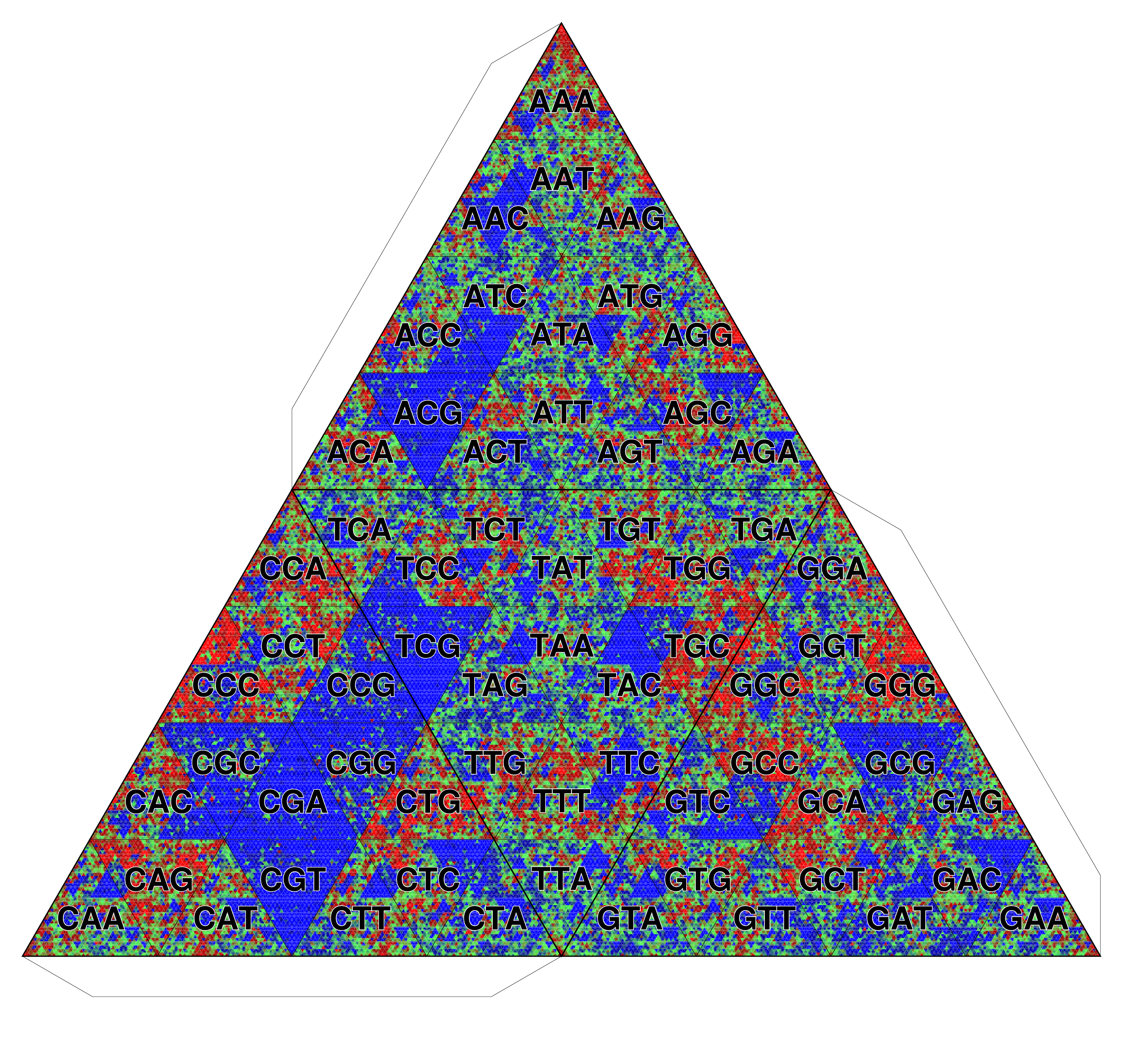

Supplement: Figure S4 — TGC of frog genome ( Xenopus tropicalis ). Octamer frequencies are depicted. The background frequency is determined by the zeroth-order Markov model. (TIFF) [file pone.0086133.s004.tiff]

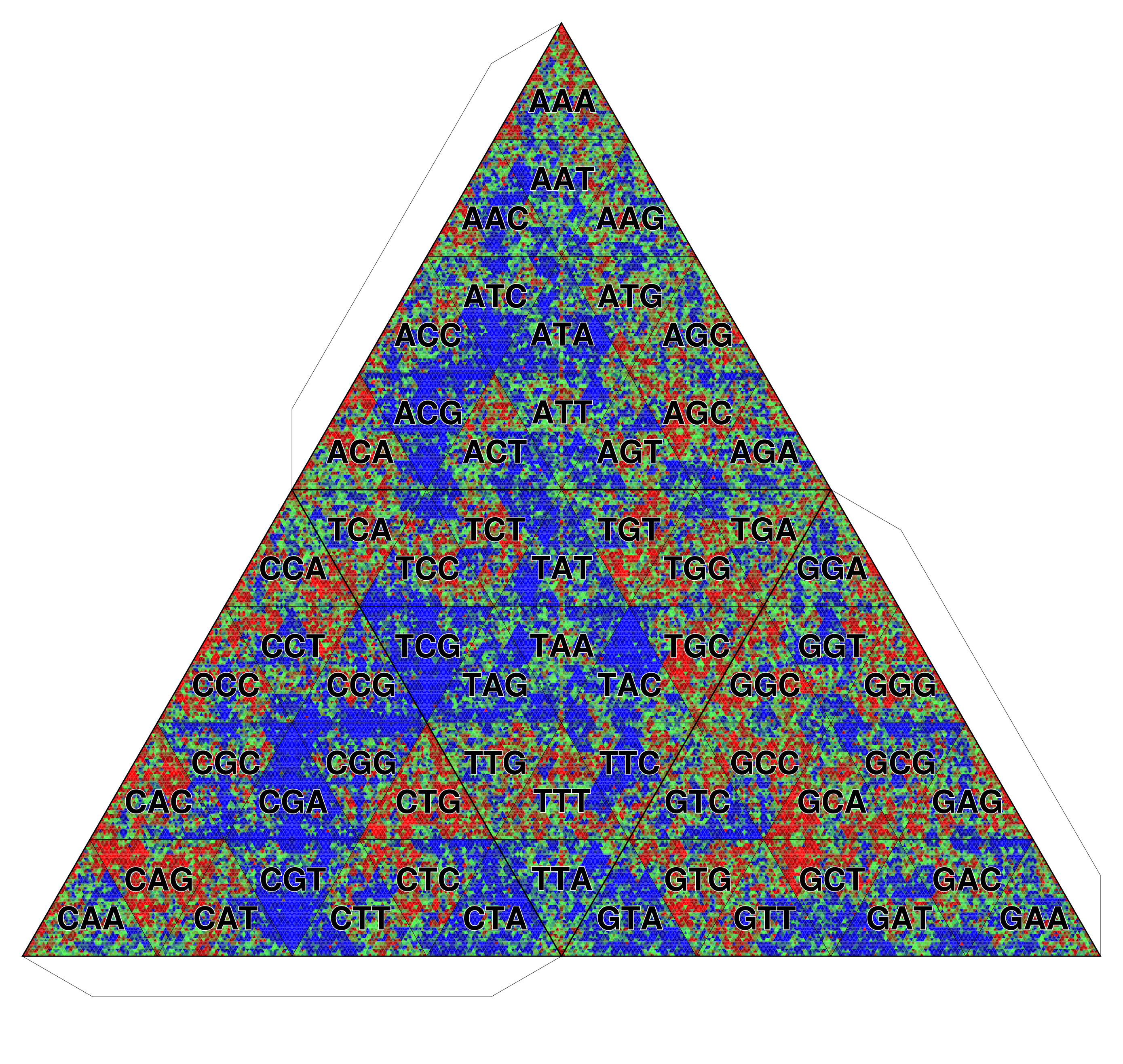

Supplement: Figure S5 — TGC of zebrafish genome ( Danio rerio ). Octamer frequencies are depicted. The background frequency is determined by the zeroth-order Markov model. (TIFF) [file pone.0086133.s005.tiff]

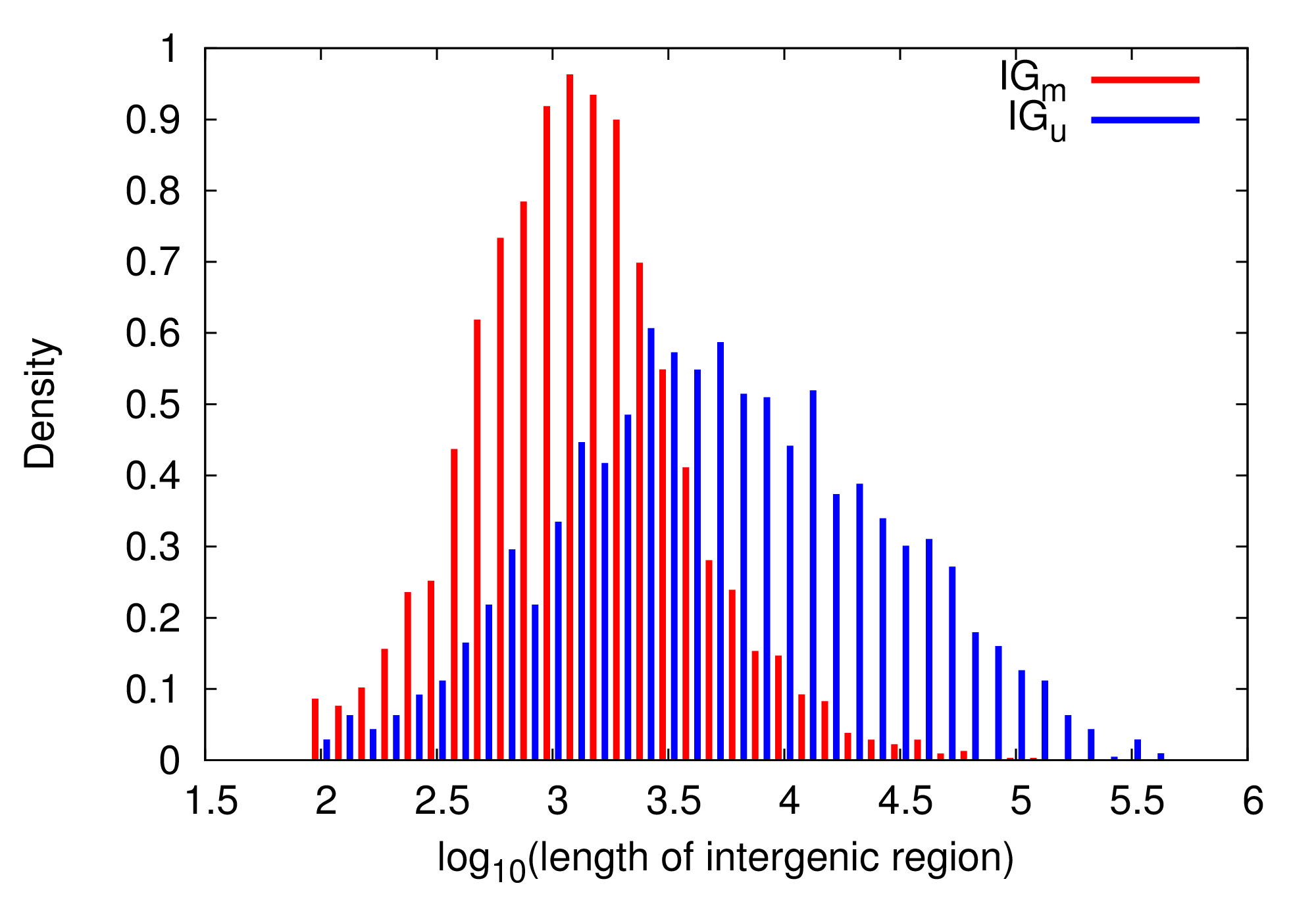

Supplement: Figure S6 — Distributions of lengths of intergenic regions IG and IG. (TIFF) [file pone.0086133.s006.tiff]

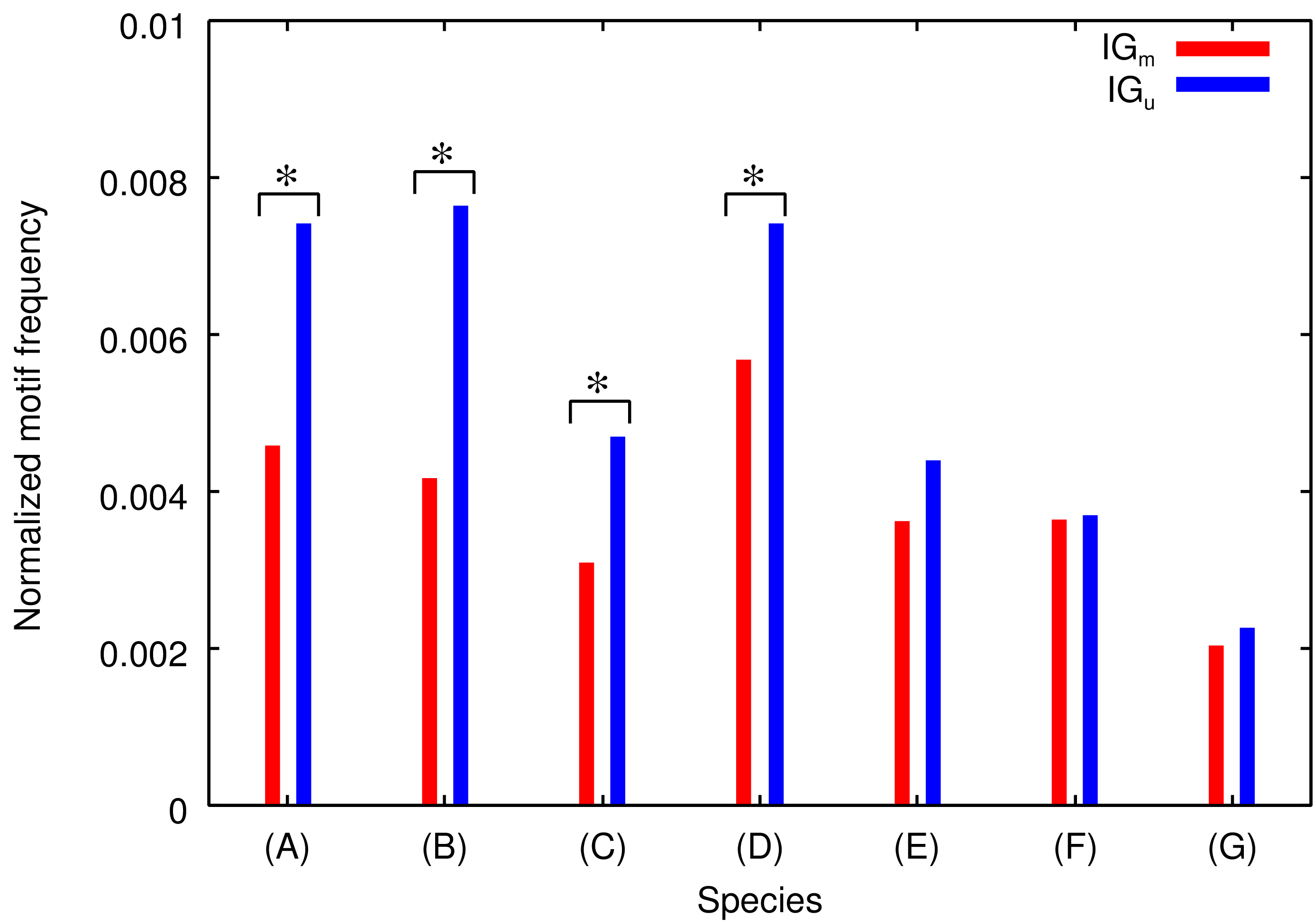

Supplement: Figure S7 — Normalized motif frequencies of CTCGAG for seven insects. (A) honey bee, (B) dwarf honey bee, (C) buff-tailed bumblebee, (D) jewel wasp, (E) red imported fire ant, (F) silkworm, and (G) pea aphid. The asterisks imply that the motif is significantly enriched in IG (significance level: ). (TIFF) [file pone.0086133.s007.tiff]

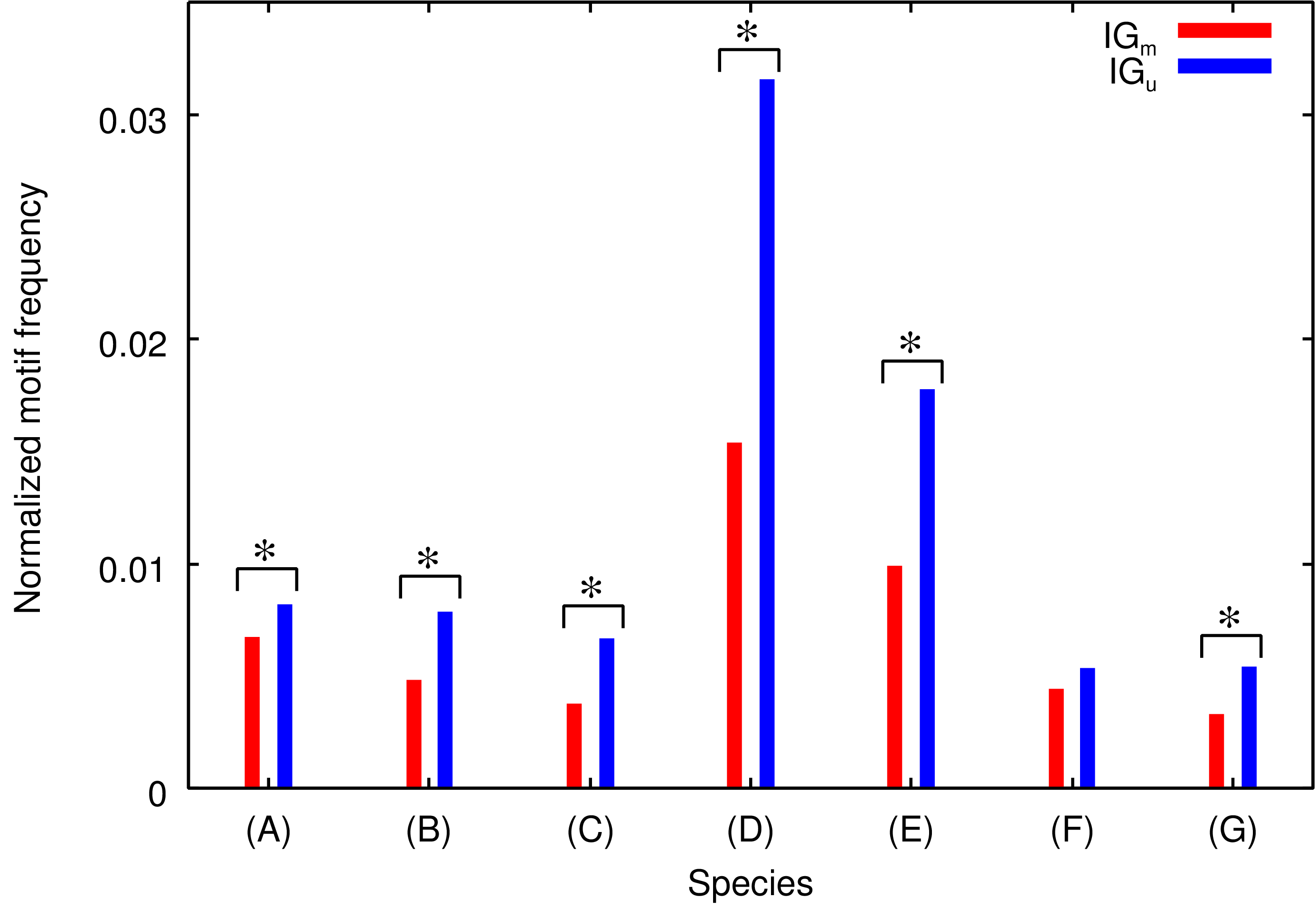

Supplement: Figure S8 — Normalized motif frequencies of CGCGCG for seven insects. (A) honey bee, (B) dwarf honey bee, (C) buff-tailed bumblebee, (D) jewel wasp, (E) red imported fire ant, (F) silkworm, and (G) pea aphid. The asterisks imply that the motif is significantly enriched in IG (significance level: ). (TIFF) [file pone.0086133.s008.tiff]
