# Supplementary material for: Tetrahedral Gray Code for Visualization of Genome Information
Source: PLoS One. 2014 Jan 27;9(1):e86133. doi: 10.1371/journal.pone.0086133 (PMC3903499; doi:10.1371/journal.pone.0086133)
Supplement: Text S1 — Details of supporting information. (PDF) [file pone.0086133.s010.pdf]

Supporting Information for: Tetrahedral Gray code for visualization of genome information

Natsuhiro Ichinose[1], Tetsushi Yada[2] and Osamu Gotoh[3]

[1]Graduate School of Informatics, Kyoto University, [2]Department of Bioscience and Bioinformatics, Kyushu Institute of Technology (KIT), and [3]Computational Biology Research Center (CBRC), National Institute of Advanced Industrial Science and Technology (AIST)

S.1 Methylation of *A. mellifera* genome

Table S1 shows the correlations of methylation in IG_m and IG_u. The intergenic regions IG_m are significantly more densely methylated than IG_u.

Fig. S6 exhibits the length distributions of IG_m and IG_u of *A. mellifera*. Because of the long tail distributions, the horizontal axis is scaled logarithmically with base 10. The average length of IG_u is significantly longer than that of IG_m.

S.2 Motif around CpG in unmethylated regions

We evaluate whether the motifs are significantly overrepresented in IG_u in comparison with IG_m. As the frequencies of CpG are different between IG_m and IG_u, we use the normalized motif frequency f_n of motif S, which is defined by:

$$f_n(S) = \frac{f(S)}{f(CG)}. \quad (1)$$

To detect the significance of the difference between IG_m and IG_u, we use the z-test and the p-value is adjusted by the number of all hexamers with CpG center (*i.e.*, 4^4). As shown in Figs. S7(A) and S8(A), the motifs of CTCGAG and CGCGCG are significantly overrepresented in IG_u in comparison with IG_m.

In Figs. S7 and S8, we evaluate whether the motifs are also significantly overrepresented in other insects. We use the following genomes related to the *A. mellifera* genome: dwarf honey bee (*Apis florea*), buff-tailed bumblebee (*Bombus terrestris*), jewel wasp (*Nasonia vitripennis*), red imported fire ant (*Solenopsis invicta*), silkworm (*Bombyx mori*), and pea aphid (*Acyrthosiphon pisum*). All of these insects are known to have DNA methylation.

Because there are no data of DNA methylation of these insects, we estimate methylated genes by their DNA contents. However, the bimodality of the CpG (o/e) ratio is not clear in some insects. Therefore, we classify genes in the two-dimensional space consisting of CpG (o/e) ratio and GC content. We apply the bivariate normal mixture model to the two-dimensional data for the genes and classify each gene into a methylated or unmethylated gene if the probability belonging to a class is greater than 0.95. In the same way as the *A. mellifera* genome, we extract IG_m and IG_u from each genome. As a result (Figs. S7 and S8), both of the motifs are significantly overrepresented in IG_u at least in related species (dwarf honey bee, buff-tailed bumblebee, and jewel wasp). CGCGCG

is significant in wider insects than CTCGAG although it is not the most prominent motif in the *A. mellifera* genome.

S.3 Choice of genome sequences

Nowadays, we can obtain a huge amount of genome sequence data that are accumulating in an exponentially growing rate. To cope with this situation, we have developed an optional tool, named GENOREP, to choose several representative genome sequences for visualizations of TGCs out of a huge set of genomes.

In this tool, a distance matrix-based clustering method is applied to the given set of genome sequences. Each pairwise distance is defined as the Kullback-Leibler divergence between k-mer frequency distributions, as we are interested in similarity of the visualized patterns rather than in the phylogenetic relationships. We implement several clustering methods that include hierarchical (single, complete, average, and Ward) clustering methods and a partitioning method (k-medoids). The number of the clusters is given by the user. The representative genome in each cluster is defined as that having the smallest average distance from the k-mer distributions of all the genomes in the cluster.

GENOREP is available at our website:
http://www.genome.ist.i.kyoto-u.ac.jp/~ichinose/padog/
